# Factors associated with unintended pregnancy, poor birth outcomes and post-partum contraceptive use among HIV-positive female adolescents in Kenya

**DOI:** 10.1186/1472-6874-12-34

**Published:** 2012-10-06

**Authors:** Francis Obare, Anke van der Kwaak, Harriet Birungi

**Affiliations:** 1 Population Council, Ralph Bunche Road, General Accident House, P.O. Box 17643, Nairobi, 00500, Kenya; 2 Royal Tropical Institute, KIT Development Policy and Practice, P.O. Box 95001, 10190, HA, Amsterdam, Netherlands

**Keywords:** HIV-positive female adolescents, Unintended pregnancy, Poor birth outcomes, Post-partum contraceptive use, Kenya

## Abstract

**Background:**

Although the experiences of unintended pregnancies and poor birth outcomes among adolescents aged 15–19 years in the general population are well documented, there is limited understanding of the same among those who are living with HIV. This paper examines the factors associated with experiencing unintended pregnancies, poor birth outcomes, and post-partum contraceptive use among HIV-positive female adolescents in Kenya.

**Methods:**

Data are from a cross-sectional study that captured information on pregnancy histories of HIV-positive female adolescents in four regions of Kenya: Coast, Nairobi, Nyanza and Rift Valley provinces. Study participants were identified through HIV and AIDS programs in the four regions. Out of a total of 797 female participants, 394 had ever been pregnant with 24% of them experiencing multiple pregnancies. Analysis entails the estimation of random-effects logit models.

**Results:**

Higher order pregnancies were just as likely to be unintended as lower order ones (odds ratios [OR]: 1.2; 95% confidence interval [CI]: 0.8–2.0) while pregnancies occurring within marital unions were significantly less likely to be unintended compared to those occurring outside such unions (OR: 0.1; 95% CI: 0.1–0.2). Higher order pregnancies were significantly more likely to result in poor outcomes compared to lower order ones (OR: 2.5; 95% CI: 1.6–4.0). In addition, pregnancies occurring within marital unions were significantly less likely to result in poor outcomes compared to those occurring outside such unions (OR: 0.3; 95% CI: 0.1–0.9). However, experiencing unintended pregnancy was not significantly associated with adverse birth outcomes (OR: 1.3; 95% CI: 0.5–3.3). There was also no significant difference in the likelihood of post-partum contraceptive use by whether the pregnancy was unintended (OR: 0.9; 95% CI: 0.5–1.5).

**Conclusions:**

The experience of repeat unintended pregnancies among HIV-positive female adolescents in the sample is partly due to inconsistent use of contraception to prevent recurrence while poor birth outcomes among higher order pregnancies are partly due to abortion. This underscores the need for HIV and AIDS programs to provide appropriate sexual and reproductive health information and services to HIV-positive adolescent clients in order to reduce the risk of undesired reproductive health outcomes.

## Background

In many parts of sub-Saharan Africa, the majority of pregnancies among adolescents aged 19 years and below result from unplanned and unprotected sexual acts and are therefore mostly unintended [1-3]. In settings with high HIV prevalence such as Eastern and Southern parts of sub-Saharan Africa, this further predisposes the adolescents to the risk of acquiring sexually transmitted infections (STIs) including HIV and AIDS. HIV infection may in turn influence the fertility of individual women, including adolescents. For example, fertility among HIV-infected women may be reduced due to diminished fecundity, increased condom use to prevent further spread of infection, or reduced sexual activity, and it may increase especially when infected individuals are under societal pressure to have sex, reproduce, or replace those children who have died [4-6]. The possible pathways through which HIV and AIDS may influence fertility could also have different implications for fertility intentions of HIV-infected women. For instance, intention to have children might be reduced for individuals who experience diminished sexual activity while it might increase for those who want to replace infants who die.

Empirical evidence indicates that in some settings, individuals who test HIV-positive reduce their childbearing intentions [7,8]. It therefore seems reasonable to suppose that in such settings, births to HIV-positive women are likely to be unintended. In other settings, the increased availability of antiretroviral treatment (ART) has been found to have a positive impact on future fertility intentions of HIV-positive individuals [9-11]. Thus, it should be expected that all factors constant, births to HIV-positive women in these settings are likely to be intended. Nonetheless, despite the increased availability of ART in parts of sub-Saharan Africa, there are reasons to suggest that unintended births might just be as common among HIV-infected adolescents as other young people in the region. For instance, recent evidence shows no significant difference in the sexual behaviour and childbearing experiences and intentions of those who were infected with HIV at birth and know their sero-status and their counterparts in the general population [12-14]. Moreover, reproductive health services in many parts of the region are not oriented towards adequately addressing the needs of adolescents partly because of weak health care systems and partly due to cultural disapproval of teenage sexuality [15-17].

Furthermore, there have been increased efforts to provide integrated reproductive health and HIV services in parts of the region as a means of improving clients’ access to both types of services [18-22]. This should in theory lead to improved reproductive health outcomes for HIV-positive clients, especially those who are on ART. However, most HIV services continue to be organized around paediatric or adult care [23,24], which implies that where the services are integrated, they rarely benefit adolescent clients. This is also supported by recent evidence indicating that for HIV-positive adolescents who have regular contacts with clinics where they can obtain sexual and reproductive health (SRH) information and services, providers/counsellors often emphasize postponement of or restraint from sexual intercourse and do not screen for SRH needs in order to offer appropriate services [13,23]. As a result, a large proportion of those who are sexually active do not use preventive methods to avoid undesired consequences such as unintended pregnancies, that is, pregnancies that occur earlier than desired (mistimed) or not wanted at all (unwanted) at the time of conception [12,13,23,25].

Unintended pregnancies have in turn been associated with low use of maternal health care services and poor birth outcomes among some population sub-groups. Marston and Cleland [26] for instance found that in Peru, children unwanted at conception had poor outcomes while in the other countries considered in the study, unintended pregnancies were associated with low use of prenatal care. There is also evidence that in many parts of sub-Saharan Africa, adolescent girls who experience unintended pregnancies resort to unsafe abortion [3,27-29]. HIV may further complicate the reproductive health outcomes of adolescents, for example, in terms of poor birth outcomes due to advanced infection especially among those who are not on ART. Moreover, experiencing repeated unintended pregnancies suggests increased exposure to unprotected sexual intercourse, which has health implications for HIV-positive individuals in terms of high risk of re-infection with another strain of the virus. Although the experiences of unintended pregnancies and poor birth outcomes among teenagers in the general population are well documented, there is limited understanding of the same among HIV-positive adolescents. This paper uses data on pregnancy histories of HIV-positive female adolescents aged 15–19 years in Kenya to examine the factors associated with experiencing unintended pregnancies, poor birth outcomes, and post-partum contraceptive use among this population sub-group.

### Context

Estimates from the 2008–2009 Kenya Demographic and Health Survey (KDHS) show that 18% of adolescent girls aged 15–19 years in Kenya had begun childbearing with marked variations by socio-economic characteristics [30]. For instance, the proportion of adolescent girls who had begun childbearing was more than twice as high in Nyanza and Coast provinces compared to Central region (27%, 26% and 10% respectively). The corresponding figures for Rift Valley, North Eastern, Western, and Nairobi provinces are 17%, 16%, 15% and 14%. In addition, the proportion of adolescent girls with no education who had begun childbearing was three times higher than that of those with secondary and above education (32% and 10% respectively). At the same time, 12% of adolescent girls were married or living with a man at the time of the survey while 1% had been formerly married (divorced or separated).

Results of the 2008–2009 KDHS further show that the national HIV prevalence among adults aged 15–49 years was 6% [30]. Prevalence was twice as high among women compared to men (8% and 4% respectively) and in Nyanza compared to Nairobi or Western provinces (14% in Nyanza compared to 7% in Nairobi and Western regions). The adult prevalence in the other regions was 5% in Rift Valley and Central, 4% in Coast and Eastern, and 1% in North Eastern province. The prevalence among adolescent boys and girls aged 15–19 years was much lower (2%) but still three times higher among girls compared to boys (3% and 1% respectively). It was also highest in Nyanza compared to other regions (6% in Nyanza compared to less than 1% in Nairobi and Eastern provinces, 1% in Central, Coast, Rift Valley and Western provinces, and 2% in North Eastern province).

Although ART was introduced in Kenya in the 1990s, the 2010 Kenya Service Provision Assessment (KSPA) found that only 16% of all health facilities were offering ART services [31]. The proportion of facilities offering the services ranged from 9% in North Eastern to 24% in Coast, 31% in Nyanza, and 33% in Nairobi partly reflecting the prevalence of HIV in these regions. Nonetheless, only 11% of the health facilities in Rift Valley offered the services despite the fact that HIV prevalence in the region is comparable to that of Coast province. The number of adults and children living with HIV in the country was estimated at 1.5 million by the end of 2009 while 61% of those in need of ART were receiving the services by the end of 2010 [32,33]. The proportion of health facilities in the country that offer HIV testing services is also high (74%) while nearly two-thirds (64%) offer care and support services for HIV-positive clients [31].

The process of integrating reproductive health and HIV services started in Kenya more than a decade ago with initial efforts focusing on integrating counselling and testing for HIV into prenatal care services [18,34]. Subsequent efforts included integrating family planning into voluntary counselling and testing for HIV as well as integrating counselling and testing into family planning services. However, it was not until 2009 that the Government finalized a national strategy for integrating the two types of services with the aim of improving the coordination and collaboration among key agencies and organizations involved in the provision of the services [34]. In spite of these efforts, the 2010 KSPA showed that of the health facilities offering prevention of mother-to-child transmission (PMTCT) of HIV services, for instance, only 33% offered the minimum package that includes testing, ART, counselling on maternal nutrition and infant feeding as well as family planning counselling or referral [31].

## Methods

### Data

The data used in this paper are from a cross-sectional study that collected information on pregnancy histories of HIV-positive adolescent girls aged 15–19 years in four regions of Kenya (Coast, Nairobi, Nyanza and Rift Valley provinces), which are characterized by high rates of HIV prevalence and teenage childbearing. The information was collected through structured interviews as part of a diagnostic study to assess the SRH information and service needs of HIV-positive adolescent boys and girls in the country. The diagnostic study was implemented by the Population Council (in Coast and Rift Valley provinces) and Plan International- Kenya (in Nairobi and Nyanza provinces) in collaboration with the partners implementing phase two of the AIDS, Population and Health Integrated Assistance program (APPHIA II partners including Family Health International- Kenya and Pathfinder International- Kenya), the National AIDS and STI Control Programme (NASCOP), and the Royal Tropical Institute (KIT) of Netherlands. Study participants were identified and recruited through the existing HIV and AIDS treatment, care and support programs with the help of service providers/counsellors. Only those who were aware of their HIV sero-status and were willing to talk about their inner lives were targeted for inclusion in the study.

The first step in the study procedures was the identification of the HIV and AIDS treatment, care and support centres in the four regions by APHIA II partners, the Nyanza Provincial Medical Officer of Health, the Nairobi Provincial Children’s Office, the District Children’s Officers- Nairobi), and the Medical Officer of Health- City Council of Nairobi. The research team then obtained permission from the management of the identified centres and held meetings with service providers/counsellors who work with the adolescents. The objective of the meetings was to introduce the study and its procedures to the providers, and to request them to identify, mobilize, and link willing participants and their respective parents/guardians with the research team after explaining to them the purpose of the study. Written parental/guardian consent and individual assent to participate in the study were obtained for respondents aged 15–17 years while individual written consent was obtained from those aged 18–19 years and those aged below 18 years who were living alone, married, or taking care of siblings. The interviews were conducted by trained young research assistants who received training on the study procedures, data collection, and ethics. The interview setting was agreed upon between the research assistants and the respondents.

A total of 1,059 adolescents completed the individual interviews with girls comprising 78% (757). Slightly more than half (394) of the girls had ever been pregnant with 24% (of 394) reporting multiple pregnancies. Those who had ever been pregnant provided detailed information on each pregnancy regarding whether it was intended, the relationship to the person responsible, prenatal care services including PMTCT, pregnancy outcome, place of delivery or pregnancy termination, assistance during delivery or pregnancy termination, post-natal care, and use of contraception after delivery or pregnancy termination. For live births, respondents were further asked whether the child was tested for HIV, their willingness to share the results of the test, the outcome of the test, and the survival status of the child. The study tools were translated into *Kiswahili* (the national language) and *Dholuo* (the dominant language spoken in Nyanza province). The study obtained ethical and research clearance from the Institutional Review Board of the Population Council, the Research Ethics Committee of the Royal Tropical Institute, the Ethics Review Committee of the Kenya Medical Research Institute (KEMRI), and the National Council for Science and Technology (NCST). Further details about the study procedures are provided in Birungi et al. [24] and Obare et al. [35].

### Statistical analysis

Analysis entails the estimation of random-effects logit models to take into account the unobserved characteristics of pregnancies or births to mothers identified from the same facility. The empirical model is given by equation (1) [36] where *π*_*ij*_ is the probability of a given outcome for pregnancy or birth *i* to mothers identified from facility *j*; *X*_*ij*_ is the vector of covariates; *β* is the associated vector of fixed parameter estimates; and *μ*_*j*_ are the unmeasured characteristics that might be correlated with the outcomes such as the socio-economic status at the time of conception, service availability and environmental factors at the time of birth as well as cultural practices guiding adolescent sexuality:

(1) $$\log it\left(\pi_{\mathrm{ij}}\right)=\beta X_{\mathrm{ij}}+\mu_{j}$$

A total of three models with dichotomous outcomes are estimated. The outcomes include experiencing an unintended pregnancy, experiencing poor birth outcomes (miscarriage, stillbirth or abortion), and using contraception after the pregnancy ended. The models include as covariates the respondents’ age at first pregnancy (in single years), study site (Coast, Nairobi, Nyanza and Rift Valley), maternal education level (no schooling, primary, and secondary and above), and paternity status (boyfriend/fiancé, husband, and friend/acquaintance/ stranger). In addition, the second and third models include whether the pregnancy was intended while the third model further includes the outcome of the pregnancy. The results are presented as odds ratios with 95% confidence intervals.

## Results

### Characteristics of ever pregnant HIV-positive adolescent girls

More than 80% of ever pregnant HIV-positive adolescent girls were aged 18–19 years (Table 1). However, most (76%) of the girls experienced their first pregnancies at the age of 17 years and below. Besides, the majority had primary-level education and came from Nyanza and Nairobi, the regions with the highest and second highest HIV prevalence in the country. About a quarter of the girls (23%) were married or living with a man at the time of the interview while 24% had been pregnant more than once. About three-quarters of the pregnancies and a similar proportion of live births were unintended.

**Table 1:** Percent distribution of ever pregnant HIV-positive female adolescents by various characteristics, Kenya

**Characteristics**	**Percent**
Current age (years)	(N=394)
15-17	17.0
18-19	83.0
Age at first pregnancy (years)	(N=394)
<15	9.4
15-17	66.5
18-19	23.6
Don’t know/missing	0.5
Education level	(N=394)
No schooling	4.3
Primary education	69.8
Secondary and above	25.1
Missing	0.8
Region	(N=394)
Nairobi	36.0
Coast	14.0
Nyanza	35.5
Rift Valley	14.5
Currently married/living together	22.8
	(N=394)
Number of times pregnant (%)^a^	(N=394)
1	75.9
2	20.3
3	3.3
4	0.5
Percentage of pregnancies that are unintended^a^	73.9
	(N=506)
Percentage of live births that are unintended	74.0
	(N=430)

^a^Include current and those pregnancies that ended in live birth, miscarriage, stillbirth or abortion.

### Experiences of unintended pregnancies

The results from the random-effects logit model predicting the likelihood of experiencing an unintended pregnancy are presented in Table 2. Pregnancies were significantly less likely to be unintended if the husband rather than the boyfriend/fiancé or other persons (friend/acquaintance/stranger) was responsible (p<0.01). There were, however, no significant variations in the likelihood of experiencing unintended pregnancy by the other characteristics considered. The lack of significant difference by pregnancy order implies that higher order pregnancies were just as likely to be unintended as lower order ones.

**Table 2:** Odds ratios from the random-effects logit models predicting the likelihood of experiencing unintended pregnancy, poor birth outcomes, and post-partum contraceptive use among HIV-positive female adolescents, Kenya

**Covariates**	**Unintended pregnancy**	**Poor birth outcomes**	**Post-partum contraceptive use**
Pregnancy order (ranges from 1 to 4)	1.2	2.5**	0.8
	(0.8 – 2.0)	(1.6 – 4.0)	(0.5 – 1.1)
Age at first pregnancy (single years)	0.9	1.1	0.9
	(0.8 – 1.1)	(0.9 – 1.3)	(0.8 – 1.1)
Study site (ref=Nyanza)			
Coast	0.8	3.1*	0.7
	(0.4 – 1.6)	(1.1 – 8.8)	(0.3 – 1.4)
Nairobi	0.9	3.1*	1.2
	(0.5 – 1.5)	(1.3 – 7.2)	(0.6 – 2.3)
Rift Valley	0.9	0.4	0.5*
	(0.5 – 2.1)	(0.0 – 3.1)	(0.2 – 0.9)
Maternal education level (ref=No schooling)			
Primary education	0.7	1.2	1.6
	(0.2 – 2.1)	(0.3 – 4.7)	(0.7 – 4.0)
Secondary and above	0.7	1.4	1.8
	(0.2 – 2.4)	(0.3 – 6.5)	(0.7 – 4.6)
Paternity status (ref=Boyfriend/ fiancé)			
Husband	0.1**	0.3*	1.4
	(0.1 – 0.2)	(0.1 – 0.9)	(0.8 – 2.5)
Other^a^	3.1	0.8	0.6
	(0.7 – 13.4)	(0.3 – 2.4)	(0.3 – 1.3)
Pregnancy was unintended (Yes=1)	n/a	1.3	0.9
		(0.5 – 3.3)	(0.5 – 1.5)
Poor birth outcome (Yes=1)	n/a	n/a	0.5
			(0.2 – 1.2)
Number of cases	496	467	453

^a^Refers to friend, acquaintance or stranger; Estimates are based on equation (1) in the text; ref: reference category; n/a: not applicable; ^*^p<0.05; ^**^p<0.01.

### Experiences of poor birth outcomes

Ten percent of the pregnancies that had ended resulted in poor outcomes, that is, miscarriage, stillbirth or abortion (not shown). Results from the random-effects logit model predicting poor birth outcomes show that higher order pregnancies were significantly more likely to result in adverse outcomes compared to lower order ones (Table 2; p<0.01). In addition, pregnancies were significantly less likely to result in adverse outcomes if they occurred within rather than outside marital unions (p<0.05). The likelihood of experiencing poor pregnancy outcomes was also significantly higher in Coast and Nairobi than in Nyanza and Rift Valley provinces. There were, however, no significant variations by the other characteristics, including whether the pregnancy was unintended.

### Post-partum contraceptive use

Respondents reported post-partum contraceptive use for 61% of the pregnancies that had ended (not shown). The results from the random-effects logit model predicting the likelihood of post-partum contraceptive use show that it was significantly lower in Coast than in Nyanza (p<0.05) or Nairobi provinces (Table 2; p<0.01). However, there were no significant variations by the other characteristics considered. It is particularly worth noting that there was no significant difference in the likelihood of post-partum contraceptive use by whether the pregnancy was unintended.

## Discussion

The objective of this article was to examine the factors associated with experiencing unintended pregnancy, poor birth outcomes and post-partum contraceptive use among HIV-positive adolescent girls in Kenya. One important finding is that HIV-positive adolescent girls who had begun childbearing experienced repeated unintended pregnancies with higher order pregnancies being just as likely to be unintended as lower order ones. The finding is consistent with emerging evidence from parts of sub-Saharan Africa that programs for HIV-positive young people have not provided pragmatic solutions for those who are or intend to be sexually active beyond emphasizing that they postpone or refrain from sexual intercourse [12,13,23,25]. The programmatic gaps have been attributed to the orientation of HIV services towards paediatric or adult care, lack of provider training in SRH counselling for HIV-positive adolescents, inadequate financial and human resources, as well as the socio-cultural challenges associated with addressing adolescent sexuality in general [23,24,35]. In spite of experiencing repeat unintended pregnancies, paternity status played an important role in whether the pregnancies were unintended with those occurring within marital unions being significantly less likely to be unintended compared to those occurring outside such unions.

The second important finding is that higher order pregnancies were significantly more likely to result in poor outcomes compared to lower order ones. This could partly be due to the use of abortion rather than contraception to space or limit births as is the case among other adolescents [3,27,28]. For instance, the significantly higher likelihood of poor birth outcomes in Coast and Nairobi provinces is consistent with the higher rates of abortion reported in these regions compared to other provinces. Estimates from the 2008–2009 KDHS show that abortion rates in Coast and Nairobi provinces were 7% compared to 2% in Nyanza or Rift Valley province [30]. Since abortion is illegal in the country, it is likely that most incidents occur under unsafe conditions thereby exposing the adolescents to greater health risks. It is, however, worth noting that paternity status was significantly associated with birth outcomes. In particular, there was a significantly lower likelihood of experiencing poor outcomes if the husband rather than other persons was responsible for the pregnancy. This suggests that adolescent girls are less likely to resort to abortion for pregnancies occurring within marital unions. In addition, unlike other studies that found an association between unwanted pregnancy and poor outcomes [26], experiencing unintended pregnancy was not significantly associated with adverse birth outcomes for the sample of adolescent girls in the study.

With respect to post-partum contraceptive use, although the adolescents in the sample reported using contraception for more than half of the pregnancies that had ended, there was no significant difference in the likelihood of use by whether the pregnancy was unintended. Available evidence suggests that although HIV-positive adolescents who receive services from treatment, care, and support programs generally report higher levels of contraceptive use compared to their counterparts in the general population, such use is inconsistent and infrequent [12-14,23]. This partly explains the occurrence of repeat unintended pregnancies among adolescents in the sample despite the relatively high levels of contraceptive use. It also implies that the frequency and consistency rather than the level of contraceptive use are important considerations for reducing unintended pregnancies among adolescents. The significantly lower likelihood of post-partum contraceptive use in Coast compared to other regions, on the other hand, partly reflects the regional variations in contraceptive use. Estimates from the 2008–2009 KDHS, for instance, show that contraceptive prevalence among currently married women is lower in Coast (34%) than in Nyanza (37%), Rift Valley (42%) or Nairobi (55%) provinces [30].

The above findings may, however, be influenced by a number of limitations. First, since the sample of HIV-positive adolescents was not randomly selected from the population, their experiences may only reflect the experiences of those who obtain services from the clinics but not of all HIV-positive adolescents in Kenya. The process of identifying and recruiting respondents was, however, necessitated by the sensitive nature of the study, hence the need to avoid exposing them to psychological or other forms of harm. Second, the study did not collect information on other factors that might affect pregnancy intentions, birth outcomes, and contraceptive use such as the individual’s socio-economic status at the time of the pregnancy, service availability and environmental factors at the time of birth as well as cultural practices guiding adolescent sexuality. It is, however, hoped that the estimation of random-effects logit models minimized the biases attributable to such unobserved characteristics. Third, it could be that the adolescent girls under-reported pregnancies that resulted in abortion, stillbirths and early neonatal deaths given the legal status of abortion in the country and the social stigma associated with such outcomes (including stillbirths and neonatal deaths). This could affect the observed associations between the various factors and birth outcomes especially if under-reporting varied by the background characteristics. Despite these limitations, the study contributes to the literature by highlighting the reproductive intentions and experiences of an understudied subset of the population.

## Conclusions

In conclusion, the experience of repeat unintended pregnancies among HIV-positive female adolescents in the sample is partly due to inconsistent use of contraception to prevent recurrence while poor birth outcomes among higher order pregnancies are partly due to abortion. The findings suggest that despite efforts to integrate reproductive health and HIV services in Kenya as a means of improving clients’ access to both services [18-20,34], HIV-positive adolescents do not seem to benefit from such integration. This could be due to the small number of facilities that provide integrated services and partly because of service orientation towards adult care. It, however, underscores the need to strengthen the provision of SRH information and services to HIV-positive adolescent clients within HIV and AIDS programs in the country in order to reduce the risk of undesired reproductive health outcomes among this population sub-group. This could be achieved through re-orienting HIV services to adequately meet the SRH needs of clients transitioning from childhood to adulthood, enhancing provider capacity through pre- and in-service training in SRH counselling for HIV-positive adolescents, and community sensitization on the SRH needs of young people living with HIV.

## Abbreviations

AIDS: Acquired immunodeficiency syndrome; APHIA: AIDS, population and health integrated assistance; ART: Antiretroviral treatment; HIV: Human immunodeficiency virus; KDHS: Kenya demographic and health survey; KEMRI: Kenya Medical Research Institute; KNBS: Kenya national bureau of statistics; KSPA: Kenya service provision assessment; NASCOP: National aids and sti control programme; NCAPD: National coordinating agency for population and development; NCST: National council for science and technology; OR: Odds ratios; PMTCT: Prevention of mother-to-child transmission (of HIV); SRH: Sexual and reproductive health; STI: Sexually transmitted infections; UNAIDS: Joint United Nations Programme on HIV/AIDS.

## Competing interests

The authors declare that they have no competing interests.

## Authors’ contributions

FO conducted the analysis, interpretation of results, and drafting of the manuscript. AK and HB were involved in the conceptual design of the study and in reviewing the manuscript for substantial intellectual content. All authors read and approved the final manuscript.

## Pre-publication history

The pre-publication history for this paper can be accessed here:

http://www.biomedcentral.com/1472-6874/12/34/prepub
